# Clinical Innovation Poster Abstracts

**DOI:** 10.1080/24740527.2019.1599264

**Published:** 2019-04-01

## Reduced Morphine Consumption, Pain Intensity, as Well as Better Early Mobilization with Local Infiltration Anesthesia versus Femoral Three-In-One Nerve Block

Michael Najfeld^a^, Robert Hube^a^, Ann-Kathrin Kujat^b^, and Kathi Thiele^b^

^a^Orthopedics, OCM, Munich, Bavaria, Germany; ^b^Orthopedics, University of Berlin Charite, Berlin, Germany

**CONTACT** Michael Najfeld michael.najfeld@ocm-muenchen.de

© 2019 The Author(s). Published with license by Taylor & Francis Group, LLC.

This is an Open Access article distributed under the terms of the Creative Commons Attribution License (http://creativecommons.org/licenses/by/4.0/), which permits unrestricted use, distribution, and reproduction in any medium, provided the original work is properly cited.

**Purpose:** We compared local infiltration analgesia (LIA) to the gold standard using the 3 in 1 nerve block in postoperative pain management after total knee arthroplasty.

**Methods:** We conducted a retrospective trial by analyzing the data of 202 patients, which were separated in two groups. Group 1 treated by 3-in-1 femoral nerve block included 100 patients whereas 102 patients were treated by LIA. The pain intensity was measured objectively with a numeric rating scale in the morning and evening. The pain medication was given according to two protocols with a fix opioid dose for the first 3 days only, additional pain medication could be requested by the patient at any time.

**Results:** The pain intensity showed statistical difference between the groups, with the LIA group showing a lower pain intensity in the early postoperative phase, especially in the first days and in the morning. On the 1^st^ postoperative day the average of numeric pain scale for the nerve block group was 2,3 (SD = 1,6), which was significant higher than the average of the LIA group 1,5 (SD = 1,1). The average dose of Oxycodon received on the first postoperative day was 17,8mg (SD = 9,1) in the nerve block group and 11,5mg (SD = 6,2) in the LIA group, on the 6^th^ day the dosage received was 10,9mg (SD = 11,3) respectively 6,0mg (SD = 7,3).

**Conclusion:** The LIA group showed in this study a significant lower consumption dose of opioid then the 3 in1 nerve block group.

## Computer-Based Application Exploring the Role of Phantom Limb “Telescoping” in Post-Amputation Pain

Andrea Aternali^a^, and Joel Katz 0000-0002-8686-447X^a^

Psychology, York University, Toronto, Ontario, Canada

**CONTACT** Andrea Aternali aternali@yorku.ca

© 2019 The Author(s). Published with license by Taylor & Francis Group, LLC.

This is an Open Access article distributed under the terms of the Creative Commons Attribution License (http://creativecommons.org/licenses/by/4.0/), which permits unrestricted use, distribution, and reproduction in any medium, provided the original work is properly cited.

More than 50% of people who have undergone limb amputation report significant levels of stump and/or phantom limb pain even years after amputation. Moreover, there are few effective treatments in treating such pain. Stump pain and phantom limb are well-known sequelae of amputation; however, a much less well-known phenomenon that develops in some people after limb amputation is “telescoping” of the phantom limb. “Telescoping” is defined as the experience of the phantom hand or foot gradually approaching the stump over time. It has been estimated to occur in approximately one third of amputees yet there is no consistent or standardized measure of the phenomenon. Clinicians and researchers have drawn attention to the need to further understand its relationship to post-amputation pain. As a result, a new computer-based application (app) evaluating telescoping in amputees has been developed using Adobe Flash animation to reliably explore its association to phantom limb and stump pain. The app allows the participant to identify the limb and level of amputation, the nature of the phantom limb, and the extent of telescoping (from normal length to completely telescoped). In addition, it collects information related to participants’ age, sex, date of amputation(s), and intensity of their stump and/or phantom pain on average on a numeric rating scale using a slider that moves from 0–10. This tool is intended to fulfill the need for a reliable measure of telescoping which can be used to document its prevalence and further understand its implications for the optimal management of phantom limb and stump pain. A fully-functional demo version of the app can be found at http://phantomlimbs.ca/demo.html

## Opioid Crisis Response: Buprenorphine/Naloxone Quality Improvement Projects in an Academic Tertiary Pain Clinic

Joyce Lee^a^, Naomi Steenhof^a^, and John Flannery^a^

Toronto Rehabilitation Institute, Comprehensive Integrated Pain Program, Toronto, Ontario, Canada

**CONTACT** Joyce Lee joyce.lee@uhn.ca

© 2019 The Author(s). Published with license by Taylor & Francis Group, LLC.

This is an Open Access article distributed under the terms of the Creative Commons Attribution License (http://creativecommons.org/licenses/by/4.0/), which permits unrestricted use, distribution, and reproduction in any medium, provided the original work is properly cited.

**Introduction/Aim:** Evaluation of the quality care outcomes from a buprenorphine/naloxone (bup/nal) protocol initiated in an outpatient chronic pain clinic using Plan-Do-Study-Act (PDSA) cycle.

**Methods:** Two cycles were completed. Cycle 1 focused on protocol feasibility. Eligible patients included individuals with chronic pain who were taking <200 mg morphine equivalents (mEq) daily, were experiencing tolerance and/or side effects to their prescribed opioids, and were unsuccessful in decreasing their opioid intake after at least one weaning attempt. Patients were seen for a total of six visits. Outcome tools included the Brief Pain Inventory (BPI), Clinical Opiate Withdrawal Scale (COWS), and QI patient survey. The QI survey explored the effects of bup/nal on quality of life (QOL), effectiveness of pain control, cravings, withdrawals, and process of care.

**Results:** Cycle 1, (N = 4, 1 weaned off prior to bup/nal; 3 successfully completed protocol), allowed cycle 2 (N = 21; 4 declined, 11 completed, 6 pending) to focus on an increase in the mEq cut-off and increasing the number of patients. Combining Cycle 1 and 2: N = 14 (7F, 7M) completed protocol; age: 27 to 74 years; average length of CP and opioid use was 15 and 10 years respectively; numerical pain score dropped by 10% and BPI functional score dropped by 16%. 84% of patients indicated that their quality of life improved and 83% rated a very positive experience with the protocol.

**Discussion/Conclusion:** A structured bup/nal protocol supports high QOL and satisfaction with process along with success and sustainability on bup/nal.

## A Framework for Biomarkers in Pain Research

Jennifer Fazzari^a^, Kimberley Begley^b^, Megan Groves^b^, and Norm Buckley 0000-0002-1031-6813^c^

^a^Department of Pathology and Molecular Medicine, McMaster University, Hamilton, Ontario, Canada; ^b^Chronic Pain Network, Hamilton, Ontario, Canada; ^c^Anesthesiology, McMaster University, Hamilton, Ontario, Canada

**CONTACT** Jennifer Fazzari fazzarjm@mcmaster.ca

© 2019 The Author(s). Published with license by Taylor & Francis Group, LLC.

This is an Open Access article distributed under the terms of the Creative Commons Attribution License (http://creativecommons.org/licenses/by/4.0/), which permits unrestricted use, distribution, and reproduction in any medium, provided the original work is properly cited.

Discussion at the Chronic Pain Network’s 2018 Annual Meeting identified biomarkers as a recurring theme in pain research, resulting in the first Canadian Consensus Conference on Biomarkers in Pain Research. Held at McMaster University, the meeting united investigators of the Chronic Pain Network involved in biomarker investigations as well as representatives from established biobanks and members of industry. Topics presented included an overview of biomarkers in pain research (Jennifer Fazzari, McMaster University); logistics of biobanking and analysis from the Canadian Longitudinal Study on Aging (Cynthia Balion, McMaster University) and the Clinical Research Laboratory and Biobank (Shana Lamers, Clinical Research Laboratory and Biobank, Hamilton), as well as commercialization of biomarkers in the chronic pain field (Brian Meshkin, Profound Ventures Inc., California, USA). Presentations by Luda Diatchenko (genetics, McGill University), Laura Stone (epigenetics, McGill University), Nader Ghasemlou (proteomics, Queens University) and Karen Davis (functional brain imaging and ethics of biomarker evaluation, University of Toronto) highlighted the challenges and potential for pain research.

In the immediate term, pain research may greatly benefit from active collaboration with other fields of research with more highly developed research infrastructure, as well as exploration of existing registries and biobanks from other fields, before embarking upon the creation of pain specific resources. The CPN Registry Working Group, and genetic analysis undertaken through the Quebec Low Back Pain project, are positioned to lead novel pain specific activities. Further discussion can be directed towards identification of a unified process for sampling, analysis and data sharing and seeking funding.

## “Smartview” Remote Monitoring and Virtual Recovery Support: A Novel Approach to Pain Postoperative Assessment and Management

Carley Ouellette^a^, Shaunattonie Henry^a^, Andy Turner^b^, Wendy Clyne^c^, Gill Furze^d^, Marissa Bird^a^, Karla Sanchez^e^, Judy Watt-Watson^f^, Sandra Carroll^g^^h^, and Michael McGillion^a^

^a^Faculty of Health Sciences, School of Nursing, McMaster University, Hamilton, ON, Canada; ^b^Faculty of Health & Life Sciences, Coventry University, Coventry, UK; ^c^Hope for the Community CIC, The Enterprise Hub, Coventry, UK; ^d^Faculty of Health and Life Sciences, Coventry University, Coventry, UK; ^e^Population Health Research Institute, Perioperative & Digital Health Department, East Hamilton, ON, Canada; ^f^Faculty of Nursing, University of Toronto, Toronto, ON, Canada; ^g^Faculty of Health Sciences School of Nursing, McMaster University, Hamilton, Canada; ^h^Perioperative & Digital Health Department, Population Health Research Institute, Hamilton, Ontario, Canada

**CONTACT** Carley Ouellette ouellc1@mcmaster.ca

© 2019 The Author(s). Published with license by Taylor & Francis Group, LLC.

This is an Open Access article distributed under the terms of the Creative Commons Attribution License (http://creativecommons.org/licenses/by/4.0/), which permits unrestricted use, distribution, and reproduction in any medium, provided the original work is properly cited.

**Background**: Coronary heart disease affects over 2.4 million Canadians annually, resulting in an increase in cardiac and major vascular surgeries. Unrelieved postoperative pain is of one of the top 5 reasons for hospital readmission following surgery; however little is done to address this postoperative complication. Barriers to effective pain assessment and management following cardiac and major vascular surgery have been conceptualized on patient, health care provider, and system levels.

**Purpose**: (1) To review commonly identified patient, healthcare provider, and system-level barriers that prevent effective postoperative pain assessment and management in cardiac and major vascular surgical populations. (2) Outline the SMArTVIEW clinical trial intervention, describing how postoperative pain is addressed in hospital, as well as 30 days post-hospital discharge by a specialized nursing team, known as SMArTVIEW Nurses.

**Methods**: By identifying existing barriers within the literature, the SMArTVIEW intervention seeks to address a number of these barriers by meeting the following design objectives: 1) orchestrating a structured process for regular postoperative pain assessment and management; 2) ensuring adequate clinician preparation for postoperative pain assessment and management in the context of virtual care; and 3) enfranchising patients to become active self-managers and to work with their healthcare providers, to manage their pain postoperatively.

**Conclusions**: SMArTVIEW is an innovative approach that has been developed to address common barriers that interdict postoperative pain assessment and management. SMArTVIEW utilizing accessible formats (i.e., digital health solutions) to actively engage both patients and healthcare providers to ensure continuity of care following hospital discharge.

## Acetaminophen Does Not Potentiate the Effect of Conditioned Pain Modulation: Results from a Double-Blind, Randomized, Crossover Trial in Healthy Participants

Yesmine Krid^a^, Guillaume Léonard^a^, and Serge Marchand^b^

^a^Surgery, Sherbrooke University, Sherbrooke, Québec, Canada; ^b^Readaptation, Sherbrooke University, Sherbrooke, Quebec, Canada

**CONTACT** Yesmine Krid yesmine.krid@usherbrooke.ca

© 2019 The Author(s). Published with license by Taylor & Francis Group, LLC.

This is an Open Access article distributed under the terms of the Creative Commons Attribution License (http://creativecommons.org/licenses/by/4.0/), which permits unrestricted use, distribution, and reproduction in any medium, provided the original work is properly cited.

Acetaminophen is widely used to reduce pain. While the mechanism of action of this pain medication is still unclear, recent observations suggest that it could involve descending inhibitory pain mechanisms. Furthermore, it seems that this mechanism might be influenced by sex, with past studies suggesting that acetaminophen potentiates inhibitory circuits differently in men and women. This study aimed to assess the effect of acetaminophen on the efficacy of inhibitory pain mechanisms in humans, and to determine whether this effect is influenced by sex. In this double-blind randomized controlled trial with a crossover design, 34 healthy volunteers (17 men, 17 women) were recruited and completed 3 experimental sessions: control (no medication), oral acetaminophen (1 g per os) and placebo (cornstarch). Conditioned pain modulation (CPM – a psychophysical measure reflecting descending pain modulating mechanisms) was assessed during each session, using heat pain stimulation (HPS – thermode) before and after conditioning stimulation (cold pressor test [CPT]); the difference in pain intensity induced by the HPS before and after CPT was used as a metric to evaluate CPM effectiveness. The pain induced by the HPS decreased significantly after the CPT, as supported by the significant main effect TIME (*p* < 0.001). However, we observed no TIME x CONDITION interaction (*p* = 0.38), indicating that acetaminophen did not affect CPM effectiveness, and no interaction with sex was noted (all *p*-values > 0.47). Altogether, these results indicate that acetaminophen does not enhance CPM effectiveness; a finding that will contribute to a better understanding and utilization of this pain medication.

## Meeting the Needs of Young Adults with Chronic Pain: A 1-Week Intensive Pain Management Program

Heather Buckingham^a^, and Laura Katz^a^

Michael G. DeGroote Pain Clinic, McMaster University Medical Centre, Hamilton, ON, Canada

© 2019 The Author(s). Published with license by Taylor & Francis Group, LLC.

This is an Open Access article distributed under the terms of the Creative Commons Attribution License (http://creativecommons.org/licenses/by/4.0/), which permits unrestricted use, distribution, and reproduction in any medium, provided the original work is properly cited.

**Introduction/Aim**: The gold standard of pain management treatment is an interdisciplinary group approach. Unfortunately, young adults (YA) often fall through the cracks of the adult healthcare system. As such, the Michael G. DeGroote Pain clinic piloted a pain management program geared specifically to meet the needs of this demographic. The aim of this poster is to describe the development and initial results of the pilot of the YA pain management program.

**Program Description:** Six Young Adults (4 females and 2 males) aged 17.9 to 20.1, attended the program. The program occurred 5 hours daily for 1 week, and consisted of participating in daily fitness, relaxation, psychoeducational classes, goal setting and functional activities. The program also included a parenting session, and a follow-up session 6 weeks post-treatment.

**Preliminary Results**: At the 6-week follow-up session, all YA reported meeting the short-term goals they set at the end of the program. Four of the six YA returned to school with the goal of obtaining college diplomas, and 2 found jobs with plans to attend college in the near future. All YA noted the benefits of connecting with other YA who struggle with chronic pain. Parents reported observing improvements in their YA’s activity levels, eating habits, independence, motivation, communication, socialization, and helping out around the house.

**Discussion/Conclusions:** The preliminary results suggest that YA benefit from engaging in group pain management programming with their peers. Future research could examine the outcomes of YA attending the regular adult group program compared to the YA only program.

## A Comparison of the Directional and Concurrent Relationships between Behavioural and Cardiac Indicators of Vaccination Pain at 12 Months

Jordana Waxman^a^, Miranda DiLorenzo^a^, Rebecca Pillai Riddell^a^, and Hartley Garfield^b^

^a^Psychology, York University, Toronto, ON, Canada; ^b^Pediatrics, University of Toronto, Toronto, ON, Canada

**CONTACT** Jordana Waxman waxmanja@yorku.ca

© 2019 The Author(s). Published with license by Taylor & Francis Group, LLC.

This is an Open Access article distributed under the terms of the Creative Commons Attribution License (http://creativecommons.org/licenses/by/4.0/), which permits unrestricted use, distribution, and reproduction in any medium, provided the original work is properly cited.

**Introduction/Aim:** The aim of this study is to provide preliminary comparative data on the relationship between healthy infants’ pain behaviour (FLACC) and commonly used cardiac indicators (i.e. heart rate [HR], respiratory sinus arrhythmia [RSA]) during 12-month vaccinations.

**Methods:** Caregiver-infant dyads were part of an ongoing cohort followed at 12-, 18- and 24-month vaccinations. Behavioural and cardiac data were simultaneously collected, and later coded/analyzed for FLACC and HR/RSA during four 30-s epochs (30-s pre-needle, immediately post-needle, 1-minute and 2-minutes post-needle). Two cross-lagged path models examined directional and reciprocal relationships between FLACC and HR or RSA during the 12-month vaccination (n = 82).

**Results:** Results indicated that: 1) Previous FLACC scores (or HR) predict future FLACC scores (or HR) across epochs (FLACC *b* = .23–1.12; HR *b* = .62-.7). Previous RSA only predicts future RSA in post-needle epochs (*b* = .22–42); 2) FLACC scores immediately (*b = *3.40) and 1-minute post-needle (*b = −1*.13) predict subsequent HR post-needle. Pre-needle FLACC scores predict RSA immediately post-needle (*b = −.2*4); and 3) Concurrent relationships between FLACC and HR were found in all but the immediate post-needle epoch (*b* = 13.86–20.55), while negative relations between FLACC and RSA were found in all epochs except 2-minutes post-needle (*b = −1*.53- [−.73]).

**Discussion/Conclusions:** Preliminary analyses suggest behaviour and cardiac indicators converge during most concurrent epochs. There were also strong indications of within-measure prediction and cross-lagged relationships between behaviour and cardiac indicators. Results suggest that behavioural and cardiac indicators may be capturing unique aspects of the nociceptive response.

## Growing Together: Development and Implementation of a Pediatric to Adult Transition Pathway in Chronic Pain Clinics across Ontario

Sarah Sheffe^a^, Jennifer Tyrrell^b^, Kyle Vader^c^, Heather Buckingham^d^, and Annette Guillemette^e^

^a^Women’s College Hospital, Toronto Academic Pain Medicine Institute, Toronto, ON, Canada; ^b^Department of Anesthesia and Pain Medicine, The Hospital for Sick Children, Toronto, ON, Canada; ^c^Kingston Health Sciences Centre, Chronic Pain Clinic, Kingston, Ontario, Canada; ^d^McMaster University Medical Centre, and Michael G. DeGroote Pain Clinic, Hamilton, ON, Canada; ^e^Ministry of Health and Long Term Care, Health Services Branch, ON, Canada

**CONTACT** Sarah Sheffe sarah.sheffe@wchospital.ca

© 2019 The Author(s). Published with license by Taylor & Francis Group, LLC.

This is an Open Access article distributed under the terms of the Creative Commons Attribution License (http://creativecommons.org/licenses/by/4.0/), which permits unrestricted use, distribution, and reproduction in any medium, provided the original work is properly cited.

An important component of healthcare transition is the coordinated movement of adolescents and young adults (AYA) with chronic medical conditions from pediatric to adult-oriented health care, leading to secure attachment to adult health services^1^. Transition is more than sending and receiving a referral: it is an explicit process that includes organizational processes, AYA preparation, transfer, and attachment to the adult care setting^2^. Chronic pain is defined as pain lasting longer than three months or beyond usual expected healing time^3^. Research has shown that, without preparation, AYA with conditions such as chronic pain are often unable to name their relevant medical history or prescriptions, adherence to care is lower, and medical complications increase^4^. Discontinuity of care is also common, leading to diminished quality of life for AYA^4^. A standardized approach to transition in care for adolescents must define responsibilities and roles for family, providers, and clients in order to provide AYA with the support necessary to achieve an optimal transition to adult care^5^. To address this need, pediatric and adult chronic pain clinics, as well as client and family partners, from across the province of Ontario collaborated to develop recommendations for transition of AYA from pediatric to adult chronic pain services. These recommendations are a starting point to support policy development, advocacy, and improved service delivery. This poster describes the guidelines created, adult and pediatric partnerships to develop and implement transition programs, and client experiences of transition from pediatric to adult chronic pain services.

### With contributions from:

Sharleen Friedman, Director of Strategy, Department of Anesthesia and Pain Medicine, The Hospital for Sick Children

Heather Boynton, Program Manager, Thunder Bay

Justina Marianayagam, patient representative

Khush Amaria, Clinical Psychologist, Good 2 Go Transition Program, The Hospital for Sick Children

Ian Bladon, Occupational Therapist, McMaster Children’s Hospital

Roxanne Krystia, Clinical Manager of Ambulatory Clinics, Sudbury Health Sciences North

Patricia Poulin, Clinical Psychologist, Ottawa Hospital

Sandra Stiles, parent representative

Susan Ward, Social Worker, The Ottawa Hospital

Jessica Wilson, Program Coordinator, Physiotherapist, CHEO

Alex Sirois, patient representative

References1.
CAPHC. Transition guidelines. 2016. accessed
https://ken.caphc.org/xwiki/bin/view/Transitioning+from+Paediatric+to+Adult+Care/Transition+Tools+and+Resources2.
Dreyer
T. Care transitions: best practices and evidence-based programs, centre for health care research and transformation. 2014.10.1097/NHH.0000000000000069248026023.
Merskey
H, Bogduk
N. Classification of chronic pain. 2nd ed. 
Seattle: 
IASP Press; 1994.4.
Stinson
J, Kohut
SA, Spiegel
L, White
M, Gill
N, Colbourne
G, Sigurdson
S, Duffy
KW, Tucker
L, Stringer
E, et al.
A systematic review of transition readiness and transfer satisfaction measures for adolescents with chronic illness. Int J Adolesc Med Health. 2014;26(2):159–74. doi:
10.1515/ijamh-2013-0512.238284885.
Clarizia
N, Chahal
N, Manlhiot
C, Kilburn
J, Redington
A, McCrindle
B. Transition to adult health care for adolescents and young adults with congenital heart disease: perspectives of the patient, parent and health care provider. Can J Cardiol. 2009
Sep;25(9):e317–e322. doi:
10.1016/S0828-282X(09)70145-X.19746251PMC2780894

## Innovation and Design of a Technology-Enabled Self-Management Curriculum to Support Pain Management and Recovery Following Cardiac Surgery

Shaunattonie Henry^a^, Carley Ouellette^a^, Andrew Turner^b^, Wendy Clyne^b^, Eric Romeril^c^, Tracy Hutchings^c^, Sarah Sharpe^d^, Paul Ritvo^e^, Michael McGillion^a^

^a^School of Nursing, McMaster University, Hamilton, Ontario, Canada; ^b^Coventry University, Coventry, UK; ^c^Hamilton Health Sciences, Hamilton, Ontario, Canada; ^d^QoC Health, Toronto, Ontario, Canada; ^e^York University, Toronto, Ontario, Canada

**CONTACT** Shaunattonie Henry henrys1@mcmaster.ca

© 2019 The Author(s). Published with license by Taylor & Francis Group, LLC.

This is an Open Access article distributed under the terms of the Creative Commons Attribution License (http://creativecommons.org/licenses/by/4.0/), which permits unrestricted use, distribution, and reproduction in any medium, provided the original work is properly cited.

**Introduction:** Educating patients following cardiac surgery has become a challenge for healthcare providers given the need to deliver complex information, limited resources, the shortened length of hospital stay, and the shift in healthcare model delivery to community-based care. As a result, patients are ill-prepared to self-manage their postoperative pain and other postoperative complications following cardiac surgery.

**Aim:** To design an e-Health curriculum, SMArTVIEW Restore and Recover (R&R), which combines postoperative education and self-management training to optimize pain and related recovery outcomes following cardiac surgery in Canada and the United Kingdom.

**Method:** Using a health engagement platform, customized modules were co-designed into an interactive digital solution. As an innovation approach, an iterative user-centered framework featuring participatory design was used to develop the web-based solution optimized for a tablet. Participants will engage in R&R, a virtual interactive self-management curriculum delivered weekly over a 5-week postoperative period. The curriculum is based on seniors needs, identified during patient journey mapping and previous self-management experience. Both content and process elements of R&R are grounded in the fear avoidance beliefs model, which shows how catastrophic pain perceptions can lead to fear, hypervigilance, avoidance, disability and depression. The curriculum is designed to provide patients with requisite cognitive, emotional, and behavioural skills to manage their postoperative pain experience in a productive and positive way, leading to optimal functioning and recovery.

**Conclusion:** SMArTVIEW R&R self-management curriculum is designed to empower seniors to communicate their postoperative pain experience and utilize effective pain management strategies. It is currently deployed as a core component of an international randomized-controlled-trial.

## Interdisciplinary Community-Based Pain Program Funded by the Ontario Ministry of Health (MOHLTC): Demographics, Pain Characteristics and Outcomes

Angela Mailis^a^^b^, Karen Spivak^a^, Jordan Robinson^a^, and S. Fatima Lakha^a^

^a^Pain and Wellness Centre, Vaughan, Ontario, Canada; ^b^Department of Medicine, Division of Physical Medicine, U of Toronto, Toronto, Ontario, Canada

**CONTACT** Angela Mailis angela.mailis@uhn.ca

© 2019 The Author(s). Published with license by Taylor & Francis Group, LLC.

This is an Open Access article distributed under the terms of the Creative Commons Attribution License (http://creativecommons.org/licenses/by/4.0/), which permits unrestricted use, distribution, and reproduction in any medium, provided the original work is properly cited.

**Introduction/Aim**: To describe the demographics, pain characteristics and effectiveness of an interdisciplinary community-based pain program, funded by the Ontario Ministry of Health and Long Term Care (MOHLTC).

**Methods**: This retrospective study was conducted on 121 well selected chronic pain patients who completed a 3–4 month intense customized interdisciplinary pain program during 2017–18. Data collected included demographic information, pain characteristics, emotional/functional status obtained by validated instruments and Global Impression of Change (GIC). Means of pre-and post-program variables were compared to assess effectiveness of each patient’s “journey”.

**Results**: Male/female ratio was 1:2 (p < 0.5); mean age 47 ± 17 years (18–85 yrs); Canadian-born patients constituted 68% (p < 0.05); 49% were employed; 28% consumed marijuana over the past year and 1/3 had received multiple injections in the past. Pain originated from MVAs, work and sports injuries in 50% of patients. Mean pain ratings and pain duration were 6.1 ± 1.6 and 5 ± 6 years respectively. Outcome data indicated substantial improvement (48% in self-efficacy, 44% in pain catastrophizing, 44% in GAD, 33% in BPI pain severity and 43% in BPI interference score, 56.5% in CESD and 79% much improved in Global Impression of Change). Detailed further analysis and 6 and 12-month outcome data will be presented.

**Discussion/Conclusions**: Our high rate of success results from: Strict inclusion/exclusion criteria for patients unable to afford the program; patient-centered one-to-one care; strong interdisciplinary communication and treatment coordination; and regimented and highly structured program. Our experience so far has taught us that such intense programs are appropriate for select pain patients only.

## NPs Practicing at Full Scope; Educational Initiatives to Support Safe and Effective Prescribing of Controlled Drugs and Substances

Elizabeth Logan^a^, and Kathy Popovski^a^

Acute Pain Service, St. Michael’s Hospital, Toronto, ON, Canada

**CONTACT** Elizabeth Logan logane@smh.ca

© 2019 The Author(s). Published with license by Taylor & Francis Group, LLC.

This is an Open Access article distributed under the terms of the Creative Commons Attribution License (http://creativecommons.org/licenses/by/4.0/), which permits unrestricted use, distribution, and reproduction in any medium, provided the original work is properly cited.

St. Michael's Hospital is an adult trauma centre, as well as being a hub for neurosurgery, cardiovascular and critical care in downtown Toronto. In April 2017, The Ministry of Health and Long-term Care and the College of Nurses of Ontario (CNO) expanded the scope of practice of nurse practitioners (NP) to include the prescribing of controlled drugs and substances (CDS), provided they complete the CNO mandated education.  At St. Michael's Hospital, all fifty NPs completed the education and were able to practice without restrictions with respect to prescribing CDS.  Despite fulfilling the educational requirements, many NPs reported they felt they required more guidance and education to prescribe safely and effectively, particularly in the context of the opioid crisis and new prescribing guidelines. This poster will describe the process of supporting NP prescribing of CDS at St Michael’s Hospital, under the leadership of the two NPs of the acute pain service.

## The Intersection of Pain and Addiction: Evolving Clinical Pathways of an Academic Health Science Centre to Provide Coordinated Patient Care

Salima S. J Ladak^a^, Diana Tamir^a^, Jiao Jiang^a^, Simone Charles^a^, Kari Van Camp^a^, Alexander Huang^a^, Karen McRae^a^, Aliza Weinrib^a^, Karim Ladha^a^, and Hance Clarke^a^

Anesthesia & Pain Management, University Health Network, Toronto, Ontario, Canada

**CONTACT** Salima Ladak salima.ladak@uhn.ca

© 2019 The Author(s). Published with license by Taylor & Francis Group, LLC.

This is an Open Access article distributed under the terms of the Creative Commons Attribution License (http://creativecommons.org/licenses/by/4.0/), which permits unrestricted use, distribution, and reproduction in any medium, provided the original work is properly cited.

As the prevalence of opioid use disorder (OUD) in Canada increases, there is a greater demand on hospitals to provide more specialized and coordinated care to patients with OUD who require surgery or have an acute medical condition involving pain. Patients at high risk for significant opioid withdrawal or those receiving current treatment for OUD and at risk for relapse during their recovery period from surgery or acute medical condition are identified early and offered coordinated and comprehensive care. Our experience to date suggests that patients in both groups require highly individualized care and expertise from a range of hospital and community-based services in order to achieve optimal pain relief peri-operatively while minimizing harm from either abrupt opioid withdrawal or relapse due to repeated opioid exposure required to manage surgical pain. This includes the multidisciplinary team consisting of pain physicians, nurse practitioners, addiction specialists, psychologists and psychiatrists. Additionally, (1) pre-operative assessments identifying patients with opioid use disorder including risk assessment for the same (2) peri-operative analgesia including harm reduction interventions and (3) successful transition to the community and sustain effective pain management while reducing opioid-related harm are all considered. This poster will illustrate, through a process mapping diagram, the key touch points along the patient’s surgical and non-surgical admission continuum at a quaternary care facility. The illustration will identify resource requirements used sequentially at each phase of the patient’s journey, including key health care professionals and interventions at each point of the pre-operative, peri-operative and post-discharge phases.

## Reconceptualizing Medical and Research Cultures with Patients through a Humanizing Relational Approach

Richard Hovey

McGill University, Montreal, Quebec, Canada

**CONTACT** Richard Hovey Richard.hovey@mcgill.ca

© 2019 The Author(s). Published with license by Taylor & Francis Group, LLC.

This is an Open Access article distributed under the terms of the Creative Commons Attribution License (http://creativecommons.org/licenses/by/4.0/), which permits unrestricted use, distribution, and reproduction in any medium, provided the original work is properly cited.

**Introduction/Aim:** The intention of this presentation / poster is to open up a conversation about engagement of patients as partners within research projects. As such the need to change the way researchers and clinicians have learned to work with each other may not be applicable when inviting people living with chronic pain to participate. I offer from perspectives found in education and philosophy relational communication approaches that can co-create a community of researchers rather a research team.

**Methods:** A philosophical hermeneutic approach was used to interpret my personal experiences as a person living with chronic pain and as a qualitative researcher.

**Results:** Changes in how we understand how to relate to each other means reconceptualizing labeling, expectations and modes of practice.

**Discussion/Conclusions:** Humanizing research offers a genuine initiation for everyone in the research team. This presentation offers insight into how to co-create such an approach which is inclusive, relational and hospitable.

Currently within the Canadian research landscape, inclusion of patients as partners, research ambassadors have become part of the fabric for research funding; “nothing about me without me”. My recent personal experiences at pain conferences and from research team meetings as a patient or more precisely, a person living with chronic pain (PLCP), who is also an academic researcher, suggest we need to evolve a philosophy of engagement that serves both the PLCPs as research ambassadors, rather than patent partners. This *presentation* is intended to open up conversations about the role of patient experience and the interconnections needed to build strong research communities, through a consideration of a whole person care relational model. In order to meaningfully locate and describe the role of the patient within the structure of a scientific research community I turn to Merleau-Ponty who aptly described the two main perspectives from which we research as, “[t]he world and man [human-beings] are accessible through two kinds of investigations, in the first case explanatory [scientific] and in the second case reflective [philosophical]”. Suggesting, that the language and relationships the emerge and nurtured within research communities need a shared understanding derived from a relational approach rather than a business model of efficiency, experts and teams. A relational approach works toward co-creating a sense of belonging and purpose rather than mere inclusion to meet research funding application criteria. The focus of this presentation is to explore how to co-create a relational approach for researchers with people living with chronic pain.

## Living in Charge: A Young Adult Pain Management Group Pilot

Sarah Sheffe

Toronto Academic Pain Medicine Institute, Women’s College Hospital, Toronto, Ontario, Canada

**CONTACT** Sarah Sheffe sarah.sheffe@wchospital.ca

© 2019 The Author(s). Published with license by Taylor & Francis Group, LLC.

This is an Open Access article distributed under the terms of the Creative Commons Attribution License (http://creativecommons.org/licenses/by/4.0/), which permits unrestricted use, distribution, and reproduction in any medium, provided the original work is properly cited.

Health care transition is the coordinated transfer of care from pediatric-oriented to adult-oriented health care for adolescents and young adults (AYA) with chronic medical conditions, leading to secure attachment to adult health services Transition programs are a key component of healthcare for this age group, and take into account the ongoing brain development and unique needs of AYA The Toronto Academic Pain Medicine Institute (TAPMI) Young Adult Group was designed to help fill this gap in pain care in Toronto, and to offer transition-focused care for clients aged 17–25 with persistent pain. The aims of the group were to promote successful health care transition, teach self-management skills, introduce clients to Acceptance and Commitment Therapy, and teach mindful movement skills. The group was developed by TAPMI Occupational and Physical Therapists. A literature search was conducted and adolescent/young adult development literature was used to guide program development. The main outcome evaluated was change in pain acceptance using the Chronic Pain Acceptance Questionnaire – 8. Client satisfaction and needs were also evaluated. This poster showcases the content and outcomes of a unique pilot group for young adults with chronic pain.

References
Canadian Association for Paediatric Health Centres (CAPHC). (2016). A guideline for transition from paediatric to adult health care for youth with special health care needs: A national approach. Retrieved from
https://ken.caphc.org/xwiki/bin/view/Transitioning+from+Paediatric+to+Adult+Care/A+Guideline+for+Transition+from+Paediatric+to+Adult+Care

## Drawing on A Child’s Perception of Pain: A Case Report

Michael Sangster^a^, Stuart Wright^b^, G. Allen Finley^c^, and Alexis Nickerson^d^

^a^Complex Pain Team, IWK Health Centre and Dalhousie University, Halifax, Nova Scotia, Canada; ^b^Department of Pediatric Anesthesia, IWK Health Centre, Halifax, Nova Scotia, Canada; ^c^Department of Anesthesia and Psychology, Dalhousie University, Halifax, Nova Scotia, Canada; ^d^Complex Pain Team, IWK Health Centre, Halifax, Nova Scotia, Canada

**CONTACT** Michael Sangster michael.sangster@iwk.nshealth.ca

© 2019 The Author(s). Published with license by Taylor & Francis Group, LLC.

This is an Open Access article distributed under the terms of the Creative Commons Attribution License (http://creativecommons.org/licenses/by/4.0/), which permits unrestricted use, distribution, and reproduction in any medium, provided the original work is properly cited.

Pain is an embodied experience that is perceived based on a person’s body schema. Neuroimaging advances suggest maladaptive plasticity of cortical somatosensory and motor representations of the body in clinical pain presentations, resulting in potential compromise of body perception across proprioceptive, exteroceptive, and interoceptive systems. This case presents a nine-year-old female with persistent right upper extremity pain secondary to a fall on outstretched hand injury and consistent with nociplastic mechanism. On initial assessment, the patient reported significant pain (6/10). The patient was cleared of neurological and orthopedic trauma. Immediate post injury pain was localized to the lateral and distal half of the right upper arm which had subsequently spread to include the right dorso-radial aspect of the hand, posterior shoulder, cervical region, and temporal and parietal regions of the head at the time of initial visit. The patient presented with normal upper extremity range of motion, no sensory deficits, positive right upper limb neurodynamic test, and pain related functional limitations. Treatment consisted of a neural mobilization program over four sessions with resultant return to full function and pain reduction to 1/10. On intake and discharge the patient was asked to draw a self-portrait and an image analysis was conducted. The intake self-portrait showed an affected arm significantly smaller than the unaffected side. The discharge self-portrait was symmetrical, suggesting normalization of her interoceptive sensitivity which correlated with her symptom resolution. This case highlights the novel consideration of body perception disturbance in clinical assessment through patient artistic conceptualizations of self.

## OnabotulinumtoxinA and Quality of Life, Health Resource Utilization, and Work Productivity in Chronic Migraine: Interim Results from the PREDICT Study

Guy Boudreau^a^, Werner J. Becker^b^, Corrie Graboski^c^, May Ong-Lam^d^, Ian Finkelstein^e^, Suzanne Christie^f^, Meetu Bhogal^g^, and Goran Davidovic^g^

^a^Neurology, Centre Hospitalier Universitaire de Montréal, Montréal, QC, Canada; ^b^Clinical Neurosciences, University of Calgary, Calgary, Alberta, Canada; ^c^Physical Medicine, Island Health, Brentwood Bay, BC, Canada; ^d^Medicine, St. Paul Hospital, Vancouver, BC, Canada; ^e^Medicine, Toronto Headache & Pain Clinic, Toronto, ON, Canada; ^f^Neurology, University of Ottawa, Ottawa, ON, Canada; ^g^Neurology, Allergan plc, Markham, ON, Canada

**CONTACT** Guy Boudreau guypboudreau@gmail.com

© 2019 Allergan. Published with license by Taylor & Francis Group, LLC.

This is an Open Access article distributed under the terms of the Creative Commons Attribution License (http://creativecommons.org/licenses/by/4.0/), which permits unrestricted use, distribution, and reproduction in any medium, provided the original work is properly cited.

**Introduction/Aim:** Chronic migraine (CM) can impair health-related quality of life (HRQoL) and daily functioning. This study assesses long-term HRQoL in adults treated with onabotulinumtoxinA for CM.

**Methods:** Multicentre, prospective, observational study in adults naïve to botulinum toxin for CM (NCT02502123). Patients received 7 onabotulinumtoxinA treatments post-baseline/screening. This analysis reports data from 4 treatments (Tx4; ~10 months). Endpoints: Mean change (baseline to Tx4) in Migraine-Specific Quality of Life (MSQ) score (primary); healthcare resource utilization (HRU) and work productivity (secondary).

**Results:** Patients at baseline (n = 196), post-Tx2 (n = 173), and post-Tx4 (n = 137) received a mean onabotulinumtoxinA dose of 170.4 U (SD = 17.2)/session with a mean between-session interval of 13.1 weeks (SD = 1.7). OnabotulinumtoxinA treatment significantly (P < 0.0001) increased MSQ scores from baseline to post-Tx4 across all role function domains (restrictive: 36.7 vs 59.8; preventive: 51.4 vs 71.3; emotional: 38.0 vs 63.6). Percentages of patients decreased from baseline to post-Tx2 and post-Tx4 for those visiting an emergency room (17.3%; 9.3%; 6.6%), admitted to hospital (3.6%; 2.9%; 1.5%), and receiving headache-related diagnostic testing (35.9%; 15.9%; 8.1%). Percentage of patients employed at baseline (73.5%) was similar post-Tx4 (72.3%); hours worked increased slightly baseline to post-Tx4 (28.0 [SD = 15.4]; 29.4 [SD = 16.0]). Headache-related missed work hours decreased (5.9 [SD = 9.5]; 2.5 [SD = 5.9]). Patients reported less impact from headache on work productivity baseline to post-Tx4 (5.4 [SD = 2.1] vs 3.9 [SD = 2.6]) and ability to perform daily activities (6.1 [SD = 2.1] vs 4.2 [SD = 2.8]). No new safety signals were identified.

**Discussion/Conclusions:** This interim analysis demonstrated that onabotulinumtoxinA for CM improved HRQoL and work productivity and reduced HRU.

**Support:** Allergan plc, Dublin, Ireland

## Active Knowledge Translation around Pain Management in Hemophilia – A National Working Group

Norm Buckley^a^, Alfonso Iorio 0000-0002-3331-8766^b^, Shannon Jackson^c^, Greig Blamey^d^, Dawn Goodyear^e^, Tim Ireland^f^, Jennifer King^g^, Peter Leung^h^, Lori Laudenbach^i^, Jayson Stoffman 0000-0002-2096-8671^j^, Jerry Teitel^k^, and Kimberly Begley^l^

^a^Department of Anesthesia, Michael G DeGroote School of Medicine, McMaster University, Hamilton, Ontario, Canada; ^a^Health Research Methods, Evidence, and Impact, McMaster University, Hamilton, Ontario, Canada; ^b^Department of Medicine, University of British Columbia and St. Paul’s Hospital, Vancouver, British Columbia, Canada; ^c^Health Sciences Centre Winnipeg, Winnipeg, Manitoba, Canada; ^d^Medicine, University of Calgary, Calgary, Alberta, Canada; ^e^Patient Perspective Partner, Vancouver, British Columbia, Canada; ^f^Saskatchewan Health Authority, Saskatchewan Bleeding Disorders Program, Saskatoon, Saskatchewan, Canada; ^g^Department of Anesthesia, St. Michael’s Hospital, Toronto, Ontario, Canada; ^h^Nurse Practitioner, Bleeding Disorders Program, London Health Sciences Centre, London, Ontario, Canada; ^i^Department of Pediatrics and Child Health, University of Manitoba, Winnipeg, Manitoba, Canada; ^j^Department of Medicine, and St. Michael’s Hospital, Division of Hematology and Oncology, University of Toronto, Toronto, Ontario, Canada; ^k^Department of Anesthesia, McMaster University, Hamilton, Ontario, Canada

**CONTACT** Norm Buckley buckleyn@mcmaster.ca

© 2019 The Author(s). Published with license by Taylor & Francis Group, LLC.

This is an Open Access article distributed under the terms of the Creative Commons Attribution License (http://creativecommons.org/licenses/by/4.0/), which permits unrestricted use, distribution, and reproduction in any medium, provided the original work is properly cited.

The Pain Management in Hemophilia (PMiH) Working Group is the first pan-Canadian multi-disciplinary task force of patient partners, hematologists, pain experts, nurses, physiotherapists, social workers and other health professionals and researchers representing the Chronic Pain Network (CPN) and the Association of Hemophilia Clinic Directors of Canada (AHCDC)

The group met to address pain care needs, research needs, training and mentoring opportunities, and develop a knowledge translation strategy addressing pain care in the hemophilia community.

The group reviewed a scoping review of pain management in hemophilia, current best practice for pain care and established a work plan. A survey was administered at the annual meeting of AHCDC and by email to CPN affiliated pain clinics; another has been developed for patients with hemophilia.

The scoping review found little high quality clinical trials evidence for pain care in hemophilia. The surveys identified a lack of pain knowledge and training amongst hemophilia clinicians, including opioid prescribing and the role of medical cannabis, and interest in augmenting knowledge. Additional meetings to review survey results and plan next steps has led to plans for interdisciplinary health team members from AHCDC to undergo a pilot training program at the DeGroote Pain Clinic. The goal is to transfer existing experience in complex pain care from pain clinic to hemophilia clinic interdisciplinary team members, create clinical pathways, and establish referral strategies to address complex problems beyond the scope of the hemophilia clinic.

Next steps identified are surveying patients, evaluating the experience with interdisciplinary training programs, and creating online resources.

## Use of Central Sensitization Inventory in Females with Chronic Pelvic Pain

Adria Fransson, Laura Katz, and Ramesh Zacharias

**CONTACT** Adria Fransson fransson@hhsc.ca

© 2019 The Author(s). Published with license by Taylor & Francis Group, LLC.

This is an Open Access article distributed under the terms of the Creative Commons Attribution License (http://creativecommons.org/licenses/by/4.0/), which permits unrestricted use, distribution, and reproduction in any medium, provided the original work is properly cited.

**Introduction/Aim**: Central sensitization is a physiological response of the central nervous system demonstrated by hypersensitivity to both noxious and non-noxious stimuli. The Michael G. DeGroote Pain Clinic piloted a novel 8-week interdisciplinary chronic pelvic pain (CPP) Program. Highlights of the Program included pelvic floor lengthening exercises, psycho-educational classes, goal setting and mindfulness. Minimal research has evaluated central sensitization in patients with CPP, and the aim of this study was to evaluate the use of the Central Sensitization Inventory (CSI) pre and post CPP Program.

**Methods**: The CSI was completed by female patients attending their first and last day of the CPP Program, along with demographics and subjective physical and pelvic functioning. Data were analyzed using descriptive statistics and a paired sample t-test.

**Results**: Eight females completed the first CPP Program and were 33.3 ± 6.2 years old. In terms of functional characteristics, 42.9% reported a strong uncontrollable urge to urinate, 84.6% reported constipation, 84.6% reported pain during/after bowel movements, and 99.9% reported pain during/after intercourse or insertion of an object. The majority of the women reported exercising 3–5 times per week (41.7%). The mean CSI total score at admission was 85.0 ± 15.0 and 77.8 ± 17.7 at discharge. A paired sample t-test was not significant (*t*= 2.6, *p*= 0.43), although this is to be expected given the low N.

**Discussion/Conclusion**: Preliminary results of this new interdisciplinary CPP Program demonstrate a pattern of high functioning females that have moderate to high levels of central sensitization. Moreover, the change in CSI post-Program encourage further analysis.

## Impact of a Personalized Home Exercise Program for Knee Osteoarthritis Patients on Physical Examination Outcomes: A Cluster Randomized Controlled Trial

Alix Cagnin^a^, Manon Choinière 0000-0001-9593-8883^b^, Nathalie J. Bureau^c^, Madeleine Durand^d^, Neila Mezghani^a^, Nathaly Gaudreault 0000-0001-7680-4898^e^, and Nicola Hagemeister^a^

^a^Laboratoire imagerie et orthopédie, École de technologie supérieure, Montréal, Québec, Canada; ^b^Anesthesiology and Pain medicine, Université de Montréal, Montréal, Québec, Canada; ^c^Radiology, Université de Montréal, Montréal, Québec, Canada; ^d^Medicine, Université de Montréal, Montréal, Québec, Canada; ^e^Rehabilitation, Université de Sherbrooke, Sherbrooke, Québec, Canada

**CONTACT** Alix Cagnin alix.cagnin.1@ens.etsmtl.ca

© 2019 École de technologie supérieure. Published with license by Taylor & Francis Group, LLC.

This is an Open Access article distributed under the terms of the Creative Commons Attribution License (http://creativecommons.org/licenses/by/4.0/), which permits unrestricted use, distribution, and reproduction in any medium, provided the original work is properly cited.

**Introduction/Aim**: The Knee Kinesiography (Knee-KG) exam is a dynamic functional test assessing mechanical risk factors linked to knee osteoarthritis (KOA). This study aimed at determining the impact on physical examination outcomes of adding this exam to current medical management (CMM).

**Methods**: Primary care clinics were randomized in three groups: 1) CMM by primary care physicians, 2) CMM plus Knee-KG-based treatment recommendations (including a personalized home exercise program), and 3) CMM, Knee-KG-based recommendations including exercises, a self-management education session, and two follow-up supervised meetings. Primary outcomes were performances on reliable physical examination tests for KOA: 2 objective tests (quadriceps strength/30 seconds chair stand test) and 6 subjective tests (e.g. flexion contracture, visual quadriceps atrophy…). Intention-to-treat and per-protocol analyses (patients who followed the recommended exercises for 6 months) were used to assess between-group differences.

**Results**: 231 patients completed the study (Group1: 76; Group2: 73; Group3: 82). At 6-month follow-up, patients from Group3 reported statistically significant improvement on functional objective tests compared to other groups (both p < 0.001). Patients in both Knee-KG groups improved compared to Group1 on the flexion contracture test (both p < 0.05). Patients in Group3 improved more than patients in Group2 (p = 0.03). Considering only patients from Group2 (32%) and Group3 (74%) who followed the exercises, all these differences with Group1 increased. Group2 further improved on visual quadriceps atrophy assessment compared to Group1 (p = 0.02).

**Conclusions**: Results support the clinical added value of a Knee-KG exam to personalize conservative treatment strategies for KOA patients in terms of performance on objective and subjective physical tests. Reinforced adherence through education and supervised sessions further improves performance.

## Growing Together: Development and Implementation of a Pediatric to Adult Transition Pathway in Chronic Pain Clinics across Ontario

Sarah Sheffe^a^, Jennifer Tyrrell^b^, Kyle Vader^c^, Heather Buckingham^d^, and Annette Guillemette^e^

^a^Toronto Academic Pain Medicine Institute, Women’s College Hospital, Toronto, Ontario, Canada; ^b^Department of Anesthesia and Pain Medicine, The Hospital for Sick Children, Toronto, Ontario, Canada; ^c^Chronic Pain Clinic, Kingston Health Sciences Centre, Kingston, Ontario, Canada; ^d^Michael G. DeGroote Pain Clinic, McMaster University Medical Centre, Hamilton, Ontario, Canada; ^e^Health Services Branch, Ministry of Health and Long Term Care, Toronto, Ontario, Canada


**With contributions from**:Sharleen Friedman, Director of Strategy, Department of Anesthesia and Pain Medicine, The Hospital for Sick ChildrenHeather Boynton, Program Manager, Thunder BayJustina Marianayagam, patient representativeKhush Amaria, Clinical Psychologist, Good 2 Go Transition Program, The Hospital for Sick ChildrenIan Bladon, Occupational Therapist, McMaster Children’s HospitalRoxanne Krystia, Clinical Manager of Ambulatory Clinics, Sudbury Health Sciences NorthPatricia Poulin, Clinical Psychologist, Ottawa HospitalSandra Stiles, parent representativeSusan Ward, Social Worker, The Ottawa HospitalJessica Wilson, Program Coordinator, Physiotherapist, CHEOAlex Sirois, patient representative


© 2019 The Author(s). Published with license by Taylor & Francis Group, LLC.

This is an Open Access article distributed under the terms of the Creative Commons Attribution License (http://creativecommons.org/licenses/by/4.0/), which permits unrestricted use, distribution, and reproduction in any medium, provided the original work is properly cited.

An important component of healthcare transition is the coordinated movement of adolescents and young adults (AYA) with chronic medical conditions from pediatric to adult-oriented health care, leading to secure attachment to adult health services. Transition is more than sending and receiving a referral: it is an explicit process that includes organizational processes, AYA preparation, transfer, and attachment to the adult care setting. Chronic pain is defined as pain lasting longer than three months or beyond usual expected healing time Research has shown that, without preparation, AYA with conditions such as chronic pain are often unable to name their relevant medical history or prescriptions, adherence to care is lower, and medical complications increase. Discontinuity of care is also common, leading to diminished quality of life for AYA. A standardized approach to transition in care for adolescents must define responsibilities and roles for family, providers, and clients in order to provide AYA with the support necessary to achieve an optimal transition to adult care. To address this need, pediatric and adult chronic pain clinics, as well as client and family partners, from across the province of Ontario collaborated to develop recommendations for transition of AYA from pediatric to adult chronic pain services. These recommendations are a starting point to support policy development, advocacy, and improved service delivery. This poster describes the guidelines created, adult and pediatric partnerships to develop and implement transition programs, and client experiences of transition from pediatric to adult chronic pain services.

References1.
CAPHC. Transition guidelines; 2016. https://ken.caphc.org/xwiki/bin/view/Transitioning+from+Paediatric+to+Adult+Care/Transition+Tools+and+Resources.2.
Dreyer
T. Care transitions: best practices and evidence-based programs. 
Centre for Health Care Research and Transformation; Washington, USA: Home Healthcare Nurse. 2014;32(5):309–316.10.1097/NHH.0000000000000069248026023.
Merskey
H, Bogduk
N. Classification of chronic pain. 2nd ed. 
Seattle: 
IASP Press; 1994.4.
Stinson
J, Kohut
SA, Spiegel
L, White
M, Gill
N, Colbourne
G, Sigurdson
S, Duffy
KW, Tucker
L, Stringer
E, et al.
A systematic review of transition readiness and transfer satisfaction measures for adolescents with chronic illness. Int J Adolesc Med Health. 2014;26(2):159–74. doi:
10.1515/ijamh-2013-0512.238284885.
Clarizia
N, Chahal
N, Manlhiot
C, Kilburn
J, Redington
A, McCrindle
B. Transition to adult health care for adolescents and young adults with congenital heart disease: perspectives of the patient, parent and health care provider. Can J Cardiol. 2009
Sep;25(9):e317–e322. doi:
10.1016/S0828-282X(09)70145-X.19746251PMC2780894

## Establishing Linkages between a Hospital-Based Chronic Pain Clinic and Community-Based Physical Activity and Exercise Programming: Challenges, Successes, and Next Steps

Kyle Vader^a^, Tom Doulas^a^, Mary Anne Good^a^, Etienne J. Bisson^a^, and Scott Duggan^a^

Chronic Pain Clinic, Kingston Health Sciences Centre, Kingston, Ontario, Canada

**CONTACT** Kyle Vader kyle.vader@kingstonhsc.ca

© 2019 The Author(s). Published with license by Taylor & Francis Group, LLC.

This is an Open Access article distributed under the terms of the Creative Commons Attribution License (http://creativecommons.org/licenses/by/4.0/), which permits unrestricted use, distribution, and reproduction in any medium, provided the original work is properly cited.

Chronic pain affects an estimated 1 in 5 adults and is a leading contributor to years lived with disability, high healthcare costs, and lost work productivity. Systematic review evidence demonstrates that physical activity and exercise can decrease pain severity and improve physical function in adults with chronic pain. Despite the benefits, participation in physical activity and exercise is low. One potential reason for low participation is that many adults with chronic pain are not sure how to access community-based physical activity and exercise programming that is tailored to meet their unique health and well-being needs. The Chronic Pain Clinic at Kingston Health Sciences Centre (KHSC) in Kingston, Ontario is a tertiary care hospital-based service in Southeastern Ontario. In order to facilitate increased participation in physical activity and exercise, the Chronic Pain Clinic at KHSC has begun to form partnerships with municipal recreation centers, the YMCA, not-for-profit agencies, and local community-based physical activity and exercise programs. In addition to simply educating patients about community-based programs, physiotherapists within the Chronic Pain Clinic at KHSC physically go into the community to participate in programs with patients to ensure appropriate connections are formed. While it has been challenging to create linkages with programs across our entire geographic catchment area, the Chronic Pain Clinic at KHSC has been successful at forming partnerships with diverse community-based physical activity and exercise programs across the greater Kingston area. Next steps include creating formalized referral pathways for patients regarding participation in community-based physical activity and exercise programming.

## Multicenter, Prospective, Randomized, Open-Label Study Comparing Efficacy, Safety, and Tolerability of OnabotulinumtoxinA and Topiramate in Chronic Migraine: The FORWARD Study

John F. Rothrock^a^, Aubrey Manack Adams^b^, Richard B. Lipton^c^, Stephen D. Silberstein^d^, Esther Jo^b^, Xiang Zhao^e^, and Andrew M. Blumenfeld^f^

^a^George Washington School of Medicine, Washington, DC; ^b^Allergan plc, Irvine, CA; ^c^Montefiore Headache Center, Department of Neurology, Albert Einstein College of Medicine, Bronx, NY; ^d^Jefferson Headache Center, Philadelphia, PA; ^e^Pharmaceutical Product Development, LLC, Austin, TX; ^f^The Neurology Center, Headache Center of Southern California, Carlsbad, CA

**CONTACT** John Rothrock jrothrock@mfa.gwu.edu

© 2019 The Author(s). Published with license by Taylor & Francis Group, LLC.

This is an Open Access article distributed under the terms of the Creative Commons Attribution License (http://creativecommons.org/licenses/by/4.0/), which permits unrestricted use, distribution, and reproduction in any medium, provided the original work is properly cited.

**Introduction/Aim**: To compare the effectiveness, safety, and tolerability of onabotulinumtoxinA and topiramate for chronic migraine (CM).

**Methods**: In the multicenter, randomized, parallel-group, open-label, prospective FORWARD Study (NCT02191579), patients received 155 U of onabotulinumtoxinA (3 treatment cycles) or topiramate (50–100 mg daily up to week 36). Patients who discontinued topiramate could cross over to onabotulinumtoxinA no earlier than 12 weeks. The primary efficacy outcome was responder rate (proportion of patients with ≥50% reduction in headache day frequency during weeks 29–32, recorded daily by patients). Secondary endpoints included change in headache day frequency, Headache Impact Test scores, and ≥70% responder rate. Adverse events (AEs) were monitored.

**Results**: 282 patients were enrolled (onabotulinumtoxinA, n = 140; topiramate, n = 142); mean±SD baseline headache days were similar (onabotulinumtoxinA, 22.1 ± 4.6; topiramate, 21.8 ± 4.8). 148 patients completed treatment as randomized (onabotulinumtoxinA, 85.7%; topiramate, 19.7%). Primary reasons for withdrawal were ineffective treatment (onabotulinumtoxinA, 5.0%; topiramate, 19.0%) and AEs (onabotulinumtoxinA, 3.6%; topiramate, 50.7%). 80 topiramate patients crossed over to onabotulinumtoxinA. Compared with baseline, a significantly higher proportion of patients had ≥50% reduction in headache frequency with onabotulinumtoxinA versus topiramate (40.0% vs 12.0%, respectively; adjusted odds ratio, 4.9 [95% CI, 2.7–9.1]; *P* < 0.001). All secondary endpoints were met (*P* < 0.001). Treatment-related AEs were reported by 17.3% and 69.7% of patients receiving onabotulinumtoxinA and topiramate, respectively.

**Discussion/Conclusions**: OnabotulinumtoxinA had a more favorable tolerability profile versus topiramate based on treatment-related AEs and overall discontinuations. OnabotulinumtoxinA was more effective than topiramate when imputation methods accounting for discontinuation differences were used.

**Support**: Allergan plc, Dublin, Ireland

## The TAPMI-ISAEC Story: A Tale of Two Ministry Funded Programs

Marcia Correale^a^, Raja Rampersaud^b^, and Tania Di Renna^c^

^a^Department of Orthopedic Surgery, University Health Network, University of Toronto, Toronto, Ontario, Canada; ^b^Department of Orthopedic Surgery, University Health Network, Tronto, Ontario, Canada; ^c^Women’s College Hospital, Toronto Academic Pain Medicine Institute, Toronto, Ontario, Canada

**CONTACT** Tania Di Renna tania.direnna@wchospital.ca

© 2019 The Author(s). Published with license by Taylor & Francis Group, LLC.

This is an Open Access article distributed under the terms of the Creative Commons Attribution License (http://creativecommons.org/licenses/by/4.0/), which permits unrestricted use, distribution, and reproduction in any medium, provided the original work is properly cited.

Inter-professional Spine Assessment and Education Clinics (ISAEC) is an innovative, upstream, shared-care model of care in which patients receive rapid low back pain assessments, education and evidence-based self-management plans. It is funded by the MOHLTC and is designed to decrease the prevalence of unmanageable chronic low back pain, reduce unnecessary diagnostic imaging as well as unnecessary specialist referral. Low back pain care pathways often involve the requirement of an interventional injection to treat or diagnose low back pain. Patients are given the option of trialing injection therapy in order to avoid surgery or as a component of therapy while awaiting surgery. Unfortunately, the waitlists for these procedures may be just as long as the wait times for surgery. These wait times render this important modality useless.

The Toronto Academic Pain Medicine Institute (TAPMI) is a program that received ministry funding in 2016. The program is a partnership amongst five teaching hospitals across the GTA. The program works as a hub to provide a biopsychosocial model of pain care delivery to chronic pain patients. The TAPMI partnership includes three interventional anesthesia sites that offer evidence based interventions to manage low back pain. TAPMI also has the benefits of a central triage system that allows for patients to be triaged to the shortest waitlist possible.

ISAEC and TAPMI have partnered to ensure seamless pathways for patients to obtain therapeutic or diagnostic block injection therapy. The plan is to replicate this partnership amongst other MOH funded clinics

## The Development of a Novel Interdisciplinary Chronic Pelvic Pain Program

Laura Katz^a^, Adria Fransson^a^, and Ramesh Zacharias^a^

Michael G. DeGroote Pain Clinic, McMaster University Medical Centre, Hamilton, Ontario, Canada

**CONTACT** Laura Katz katzl@hhsc.ca

© 2019 The Author(s). Published with license by Taylor & Francis Group, LLC.

This is an Open Access article distributed under the terms of the Creative Commons Attribution License (http://creativecommons.org/licenses/by/4.0/), which permits unrestricted use, distribution, and reproduction in any medium, provided the original work is properly cited.

**Introduction**: Chronic pelvic pain (CPP) is a significant issue for women, with approximately 14% of women experiencing CPP at least once in their life. CPP is chronic and debilitating associated with significant costs and morbidity, and its etiology is multifactorial often complicating medical treatment and symptom management. Best practice guidelines recommend an interdisciplinary and biopsychosocial approach to treatment. The aim of this poster is to describe the ongoing development and evaluation of a novel Interdisciplinary CPP Program at the Michael G. DeGroote Pain Clinic.

**Methods**: Female patients were referred to the Program from community gynecologists and urologists, and were scheduled for an orientation to learn about the Program. Patients were then scheduled for an interdisciplinary assessment (psychology, physiotherapy, internal pelvic examination), and if appropriate were scheduled for the Program. The Program occurs once a week for 8 weeks, and each day consists of physiotherapy, psycho-education, goal setting, and mindfulness.

**Preliminary Results & Discussion**: Ninety-four referrals have been received since January 2018. Eight out of nine patients completed the first Program, and assessments and programs are ongoing. In terms of the demographics of the first sample of patients, the mean age was 33.3 ± 6.2, they last worked 2.8 ± 2.2 years ago, their pain started 10.1 ± 7.5 years ago, with predominantly an underlying diagnosis of endometriosis (77.8%). Ninety percent reported a history of anxiety/panic and depression. Upon discharge, all outcomes measures showed improvements. As the Program develops, future research will evaluate the statistical changes in outcomes, and will continue to support women coping with CPP.

## Pain among Adolescents May be Amplified by a Difficult Family Situation and Insecure Relationships with Peers

Siv Skarstein^a^, Lisbeth Gravdal Kvarme^a^, Marit Leegaard^a^, and Sølvi Helseth^a^

^a^Faculty of Health and Science, Oslo Metropolitan University, Oslo, Norway

**CONTACT** Siv Skarstein siv.skarstein@oslomet.no Faculty of Health and Science, Oslo Metropolitan University, Postboks 4. St. Olavs plass, Oslo 0130, Norway

© 2019 The Author(s). Published with license by Taylor & Francis Group, LLC.

This is an Open Access article distributed under the terms of the Creative Commons Attribution License (http://creativecommons.org/licenses/by/4.0/), which permits unrestricted use, distribution, and reproduction in any medium, provided the original work is properly cited.

**Aim and objectives**: Frequent self-medication with analgesics among adolescents is associated with general pain, several physical pain points, low self-esteem and low ambitions for the future. The objectives of our study was to increase our knowledge about conditions affecting pain experiences, pain management and development of identity in adolescents frequently using over-the-counter analgesics.

**Design**: Qualitative individual interviews with adolescents and their mothers, analysed as dyads.

**Setting and participants**: Students aged 14–16 in 9th and 10th grade in 10 Norwegian junior high schools self-reporting at least weekly use of analgesics were asked to participate. Those who wanted to take part brought a consent letter to their parents, also inviting the parent to participate.

**Results**: Six girls, two boys and their mothers were included. The teenagers’ stories showed that they were highly dependent on their mothers. They had often been bullied, avoided conflicts and strived to be accepted. Their mothers felt solely responsible for their upbringing and showed great concern for all the pain experienced by their child. A close relationship between mother and child influenced how the adolescent managed their pain, including their use of over-the-counter analgesics. Three main themes were identified in the stories of mother and child; “Vulnerable adolescents”, “Mother knows best” and “Pain is a shared project”.

**Conclusions**: Pain and development of identity are amplified by difficult family situation and insecure relationship with friends. The adolescents learn pain management mainly from their mothers. The mothers have the main responsibility for upbringing and experience little support from family or health professorial.

## Sleep Health and the Young Adult Pain Population: An Interprofessional Approach

Mandeep Singh^a^, Sarah Sheffe^b^, Shikha Bansal^a^, and Tania Di Renna^a^

^a^Women’s College Hospital, Toronto Academic Pain Medicine Institute, Toronto, Ontario, Canada; ^b^Anaesthesia, Women’s College Hospital, Toronto, Ontario, Canada

**CONTACT** Tania Di Renna tania.direnna@wchospital.ca

© 2019 The Author(s). Published with license by Taylor & Francis Group, LLC.

This is an Open Access article distributed under the terms of the Creative Commons Attribution License (http://creativecommons.org/licenses/by/4.0/), which permits unrestricted use, distribution, and reproduction in any medium, provided the original work is properly cited.

Toronto Academic Pain Medicine Institute (TAPMI) is an interdisciplinary academic pain program serving as a hub for chronic pain in Toronto. The TAPMI Young Adult Clinic (YAC) started in 2018 as a transition program to guide paediatric chronic pain patients into the adult healthcare system. Currently, this is the only transitional aged chronic pain clinic in Ontario.

Long-term sleep disruption is associated with increased chronic pain. The degree of pain relief can directly impact the quality and disruption of sleep. Data on the association in the young adult chronic pain population and sleep is scarce.

The YAC serves patients aged 17–25 years. its core clinical team comprises of an occupational therapist, physical therapist, and chronic pain physicians. It was noticed that YAC patients had problems with their sleep health, ranging from sleep initiation, sleep maintenance, circadian disturbances, primary intrinsic disorders such as apnea or restless legs syndrome. Patients also reported significant interaction between the pain quality, pain control and medications with their sleep health and overall quality of life.

In view of our recent findings, we recently expanded this program by creating a multi-disciplinary program integrating systematic evaluation of sleep health disruption, coordinating treatment strategies in consultation with the chronic pain physician, and occupational therapist with the goal to improve overall health, and quality of life of this vulnerable patient population. Various validated subjective and objective measures of sleep health, pain condition, pain perception, self-efficacy and quality of life will be measured prospectively in this novel inter-professional clinic.

## The Implementation of Infant Pain Practice Change (ImPaC) Resource: From prototype to refined version using a user-centered design approach

Mariana Bueno^a^, Shirine Riahi^a^, Alexa Lanese^a^, Shelly-Anne Li^a^^b^, and Bonnie Stevens^a^^b^

^a^Child Health Evaluative Sciences, The Hospital for Sick Children, Toronto, Ontario, Canada; ^b^Lawrence S Bloomberg Faculty of Nursing, University of Toronto, Toronto, Ontario, Canada

**CONTACT** Mariana Bueno mariana.bueno@sickkids.ca

© 2019 The Author(s). Published with license by Taylor & Francis Group, LLC.

This is an Open Access article distributed under the terms of the Creative Commons Attribution-NonCommercial License (http://creativecommons.org/licenses/by-nc/4.0/), which permits unrestricted non-commercial use, distribution, and reproduction in any medium, provided the original work is properly cited.

**Introduction/Aim**: To apply the user-centered design (UCD) approach to evaluate the ImPaC Resource.

**Methods**: UCD principles were used in prototype development (Phase 1) and refinement (Phase 2), to evaluate its usability by health care professionals (HCP). In Phase 1, (i) 9 HCP evaluated content adequacy, (ii) 8 HCPs evaluated usability, and (iii) 80 HCP webinar participants evaluated feasibility and appropriateness. In Phase 2, (iv) 10 HCP navigated the prototype in “think-aloud” semi-structured interviews using a prescribed scenario in a nonclinical setting, and (v) 4 HCPs implemented the prototype for 4 months in a single NICU and participated in a focus group interview.

**Results**: Phase 1: (i) 9 HCP strongly agreed the Resource adequately represented pain practice change; a content validity index > 0.8 supported components relevance; (ii) 8 HCPs agreed the Resource had acceptable form and content; and, 86% of the 80 webinar participants agreed it was feasible and clinically appropriate. Phase 2: (iv) 10 HCPs reported the Resource prototype was intuitive, easy to navigate, appealing, and evidence-based; (v) 4 HCP identified design requirements including individual login structure, improved visual representation of the workflow and connections, additional visual representation of clinical data, enhanced functionality for planning and evaluation, and optimization for common tasks.

**Conclusions**: The UCD approach enabled the redesign and refinement of a scaled-up prototype based on evaluation from 5 sets of users. The refined Resource will undergo final usability testing before launching a nationwide trial to evaluate its effectiveness in clinical settings.

## A Patient Informed Qualitative Program Evaluation of an Internet-Based Chronic Pain Treatment

Pamela L. Holens^a^, Adair Libbrecht^b^, Michelle Paluszek^c^, Alyssa Romaniuk^b^, Brent Joyal^b^, Jeremiah Buhler^b^, and Luigi Imbrogno^d^

^a^Clinical Health Psychology, University of Manitoba, Winnipeg, Manitoba, Canada; ^b^Psychology, University of Manitoba, Winnipeg, Manitoba, Canada; ^c^Psychology, University of Regina, Regina, Saskatchewan, Canada; ^d^Biology, University of Manitoba, Winnipeg, Manitoba, Canada

**CONTACT** Pamela L. Holens pholens@deerlodge.mb.ca

© 2019 The Author(s). Published with license by Taylor & Francis Group, LLC.

This is an Open Access article distributed under the terms of the Creative Commons Attribution License (http://creativecommons.org/licenses/by/4.0/), which permits unrestricted use, distribution, and reproduction in any medium, provided the original work is properly cited.

An innovative, Internet-based chronic pain treatment tailored to a military and police population was developed using Acceptance and Commitment Therapy (ACT) as a model. The treatment was recently evaluated in randomized controlled trial and found to be superior to treatment as usual in terms of increasing patients’ levels of pain acceptance, decreasing their pain-related catastrophizing, and decreasing their levels of kinesiophobia. In an effort to further increase the efficacy of the treatment, we enlisted patient feedback about the program through a series of focus groups. Participants who had previously completed the online treatment were recruited to participate in a series of focus groups designed to qualitatively evaluate the treatment and offer suggestions for improvements for future versions of the program. Participatory Action Research methodology was used to conduct this study and data were examined using interpretive thematic analysis. Three main themes arose: suggestions for improving the technological “friendliness” of the online program, suggestions for improving the sequencing of content, and suggestions for greater tailoring of the content to the sensitivities of the target population. As an example of the latter, participants suggested removal of the “attending your own funeral” exercise from the values module due to sensitivities around death and dying. Future directions, based on patient feedback, are outlined.

## Quantitative Sensory Testing Influences Treatment in Paediatric Chronic Pain Interdisciplinary Clinic

Alice Bruneau^a^, Marta Somaini^b^, Nada Mohamed^b^, Pablo M. Ingelmo^b^, and Catherine E. Ferland^c^

^a^Integrated Program in Neuroscience, McGill University, Montreal, Québec, Canada; ^b^Chronic Pain Service, Montreal Children’s Hospital, Montreal, Québec, Canada; ^c^Department of Anaesthesia, McGill University, Montreal, Québec, Canada

**CONTACT** Alice Bruneau alice.bruneau@mail.mcgill.ca

© 2019 The Author(s). Published with license by Taylor & Francis Group, LLC.

This is an Open Access article distributed under the terms of the Creative Commons Attribution License (http://creativecommons.org/licenses/by/4.0/), which permits unrestricted use, distribution, and reproduction in any medium, provided the original work is properly cited.

**Introduction:** There is currently no strong evidence to use pharmacological treatments in paediatric chronic pain. Quantitative sensory testing (QST) provides information on the mechanism of pain, which may help clinicians to choose the appropriate treatment. Our objective was to evaluate the relevance of QST as a mechanism-based therapeutic targeting tool in a paediatric chronic pain clinic.

**Methods:** Patients were assessed using QST for the presence of peripheral (PS) and central sensitization (CS). Their endogenous inhibitory pain control efficacy was evaluated using a conditioned pain modulation (CPM) paradigm. The information was provided to the health care professional before the initial visit. The primary end-point was the proportion of patients receiving pain medication.

**Results:** 165 patients (62 with QST assessment and 103 controls, mean age 14.5 ± 2.2 years old) were analysed retrospectively. Differences in medical treatment were observed between the QST group and the control group using Fisher’s Exact Test. QST patients received less opioids than controls (9.7% vs 29.1% respectively, P = 0.003) and less anticonvulsants (5.3% vs 16.2%, P < 0.001). Among patients with a chronic secondary pain diagnosis (including musculoskeletal, neuropathic and posttraumatic/postsurgical pain), this difference was even more noticeable for both opioid prescriptions (10% vs 40.8%, P = 0.04), and anticonvulsants (0% vs 34.7%, P < 0.001). Furthermore, patients who had an optimal inhibitory pain control were prescribed less antidepressants (29.4% vs 3.8%, P = 0.016).

**Conclusions:** Using QST assessments reduced the use of medication, suggesting its valuable use to guide treatment in the daily clinical practice.

## Reduction of Headache Frequency in Adult Women When Addressing the Dural Fascial Kinetic Chain (DFKC)

Satya Sardonicus^a^, and Danielle Ayres^a^

Private Practice of Chiropractic, Portland, OR, USA

**CONTACT** Satya Sardonicus drsatyasardonicus@gmail.com

© 2019 The Author(s). Published with license by Taylor & Francis Group, LLC.

This is an Open Access article distributed under the terms of the Creative Commons Attribution License (http://creativecommons.org/licenses/by/4.0/), which permits unrestricted use, distribution, and reproduction in any medium, provided the original work is properly cited.

**Introduction**: Fascia condenses in functional lines throughout the body to promote better kinetics and to cover and connect different body structures. Its purpose in the body has historically been dismissed and discarded. There is currently little research on the connection between fascial lines in the lower extremity to the dural sleeve, and its implications in clinical care. The purpose of this research is to examine how a treatment protocol focused on decreasing posterior lower line fascial tension and dural tension improves the symptomatology of patients suffering from headaches

**Methods**: We performed retrospective file review (2015–2018) of female adults presenting for care with headaches. Inclusion criteria for review were: (1) the patient underwent a full chiropractic examination prior to care; (2) the patient received consistent care consisting of spinal adjustments and fascial release within a NeuroFascial Integration framework.

**Results**: We found 7 patients meeting inclusion criteria. Their average age was 42 years. All seven experienced decreased frequency of their headache symptoms. Dural tension and lower posterior fascial line tension objectively improved using the orthopedic measures tested.

**Conclusion**: Our findings provide supporting evidence that spinal adjustments and fascial release may improve dural tension. We support future research on the non-invasive ways to improve dural tension that may contribute to patients suffering from headaches.

## Toronto Academic Chronic Pain Medicine Institute (TAPMI): Innovations in Psychosocial Program Recruitment & Delivery

Orit Zamir^a^, Lalia Palacio^b^, Claire Richardson^b^, Wendy Carter^a^^b^, and Rachael Bosma^a^^b^

^a^Women’s College Hospital, Department of Psychiatry, Toronto Academic Pain Medicine Institute, Toronto, Ontario, Canada; ^b^Women’s College Hospital, Toronto Academic Pain Medicine Institute, Toronto, Ontario, Canada

**CONTACT** Orit Zamir Orit.Zamir@wchospital.ca

© 2019 The Author(s). Published with license by Taylor & Francis Group, LLC.

This is an Open Access article distributed under the terms of the Creative Commons Attribution License (http://creativecommons.org/licenses/by/4.0/), which permits unrestricted use, distribution, and reproduction in any medium, provided the original work is properly cited.

It is estimated that 1 in 5 Canadians experiences chronic pain, yet many studies show that pain is poorly managed despite its prevalence. In Toronto, patients can spend up to 20 months waiting to receive specialized chronic pain care. In an effort to offer streamlined and accelerated access to care, Women’s College Hospital has partnered with the Centre for Addiction and Mental Health, Sinai Health System, St. Michael’s Hospital and the University Health Network to create a hub for chronic pain (TAPMI).

Two of TAPMIs psychosocial groups are: Cognitive Behavioural Therapy (CBT) and Mindfulness Based Stress Reduction (MBSR). CBT provides strategies to address unhelpful thought and behaviour patterns, exacerbating pain and mood. Patients learn pain is not just a physical sensation – it also impacts one’s emotions, behaviours, and thoughts.

MBSR is a program that incorporates mindfulness practice which includes awareness of present moment experience through the breath, body scan, mindful movement, and walking and sitting meditations and through the cultivation of attitudes like non judgement. Research shows that both groups aim to increase qualify of life and global functioning and reduce pain-related disability e.g., psychological distress, stress.

As the TAPMI program has evolved so has the process for recruiting, assessing and registering patients to the psychosocial groups (CBT or MBSR). This poster describes the lessons learned through TAPMIs program development, expansion, quality improvement cycles, and outcomes.

## Medical Cannabis Induced Acute Pancreatitis and Hyperemesis Syndrome in a Patient with Complex Regional Pain Syndrome

Cheryl Hutflesz^a^, and Vikas Parihar^a^

McMaster University Medical Centre, Michael G. DeGroote Pain Clinic, Hamilton, Ontario, Canada

**CONTACT** Cheryl Hutflesz hutfleszc@hhsc.ca

© 2019 The Author(s). Published with license by Taylor & Francis Group, LLC.

This is an Open Access article distributed under the terms of the Creative Commons Attribution License (http://creativecommons.org/licenses/by/4.0/), which permits unrestricted use, distribution, and reproduction in any medium, provided the original work is properly cited.

**Introduction**: Medical cannabis is being utilized for intractable pain that is not well managed through conventional means. There are reports of cannabis induced hyperemesis syndrome and acute pancreatitis amongst recreational users inhaling cannabis, but not amongst medical users consuming cannabis orally

**Patient Case**: A 33-year old male with complex regional pain syndrome initiated 1 g of medical cannabis per day consisting of CBD (25 mg/mL) and tetrahydrocannabinol (<2 mg/mL). He transitioned to a balanced 50:50 CBD to THC product 12.5 mg/mL of each every six hours. He achieved good pain and anxiety relief but developed gastrointestinal cramping, bloating, intermittent nausea and vomiting which he managed with homeopathic remedies. He added THC only at bedtime and symptoms exacerbated over 3 months. He experienced worsening abdominal pain, nausea, projectile vomiting and weight loss. He was found to have epigastric and right and left upper quadrant abdominal tenderness as well as elevated lipase >300. Abdominal ultrasound and CT abdomen with contrast were negative. Serial lipase levels rose to >800. He stopped all cannabis products. Oral Haldol 0.5mg QID was initiated for hyperemesis after ondansetron failure. After 1 week CBD dominant oil was reintroduced at 1ml (25mg) QID for CRPS with no worsening of GI symptoms. After 2.5 weeks off THC hyperemesis dramatically improved, weight loss stabilized and lipase returned to baseline.

**Conclusion**: Chronic oral administration of medical cannabis containing tetrahydrocannabinol resulted in acute pancreatitis and hyperemesis similar to individuals inhaling recreational cannabis. Patients and healthcare providers should be counselled on the rare but serious complications of medical cannabis induced pancreatitis.

## Inagene Targeted Pain Panel: A New Pain-Focused Genotyping Platform to Guide Personalized Pain Management Decisions

Ben Pinder^a^, and Kathy Siminovitch^a^

R&D Division, Inagene Diagnostics Inc., Toronto, Ontario, Canada

**CONTACT** Ben Pinder bpinder@Inagene.com

© 2019 Inagene Diagnostics. Published with license by Taylor & Francis Group, LLC.

This is an Open Access article distributed under the terms of the Creative Commons Attribution-NonCommercial License (http://creativecommons.org/licenses/by-nc/4.0/), which permits unrestricted non-commercial use, distribution, and reproduction in any medium, provided the original work is properly cited.

Pain is among the most common reasons for seeking medical attention, yet pain management remains challenging and ineffective for many patients. Poor pain management severely reduces quality of life and incurs significant socioeconomic burden, causing loss of productivity and increased healthcare costs.

Cumulative data suggests that up to 60% of the variable responses to pain medications relate to genetic polymorphisms in genes involved in the absorption, distribution, metabolism and/or excretion of these drugs. An individual’s response to pain medications and also propensity for addiction to such drugs, may therefore be significantly influenced by the composite of variants present in such genes and, by extension, knowledge of each individual genotype profile enables more informed drug/dose selection so as to more effectively alleviate pain and reduce frequency of adverse drug reactions.

To provide pharmacogenetic profiles specifically related to pain medications, Inagene Diagnostics has developed a pain treatment-focused, state-of-the art genotyping platform. Unlike other pharmacogenetics panels, the Inagene test is specifically tailored to address the needs of individuals suffering from different types of pain, comprehensively assaying the greatest number of genes/gene variants validated as having significant effects on efficacy of pain medications.

The Inagene testing service is available to prescribing physicians, pharmacists, and direct to consumers to guide healthcare providers and patients in the choice of analgesics, opioids, cannabinoids, and many other pain relief medications. This uniquely comprehensive assay serves as a novel guidance platform for difficult therapy decisions, heralding a more effective personalized approach to pain management.
